# 

**DOI:** 10.1192/bjb.2022.65

**Published:** 2023-08

**Authors:** Jan R. Oyebode

**Affiliations:** Professor of Dementia Care in the Centre for Applied Dementia Studies, University of Bradford, UK. Email: j.oyebode@bradford.ac.uk

**Figure:**
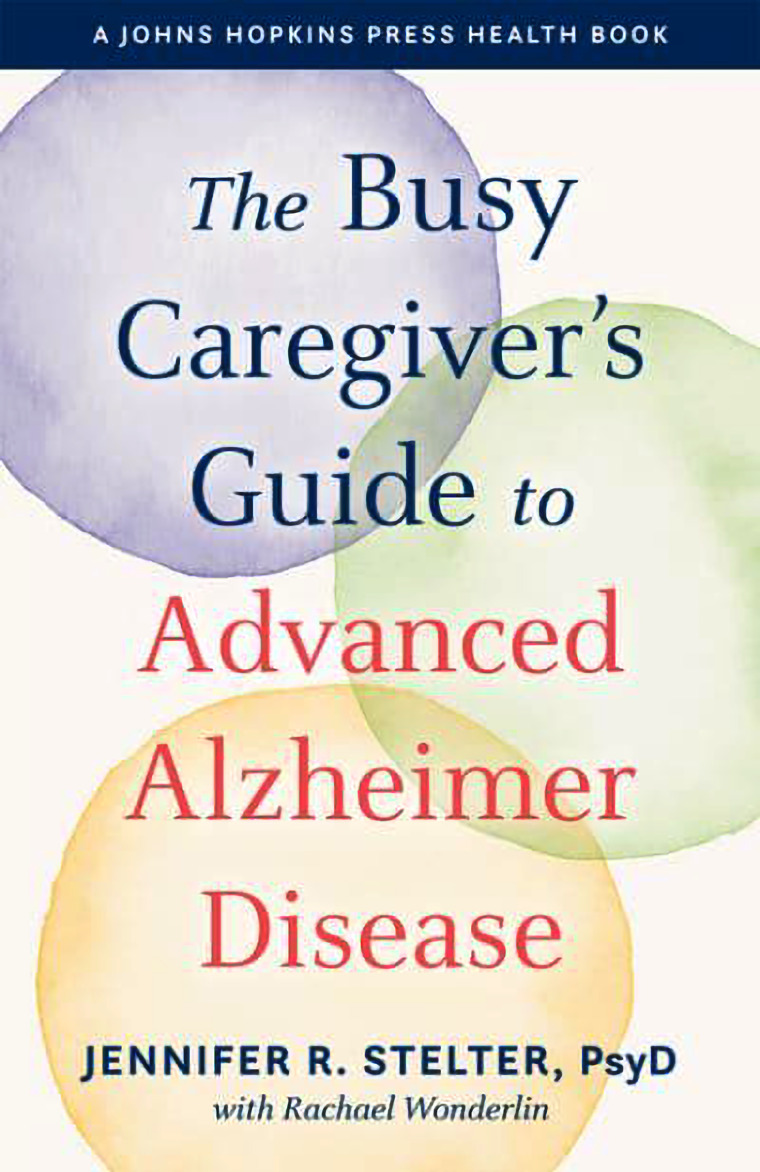

In this short book, the author offers family carers advice on how to look after a person with advanced Alzheimer's disease. She bases her writing on a ‘dementia connection model’, bringing together retrogenesis (i.e. that individuals with Alzheimer's disease are systematically regressing in developmental age, ultimately to babyhood), habilitation (i.e. that people with Alzheimer's disease learn through procedural memory) and sensory knowledge (i.e. that the emotions of people with dementia are influenced by their senses). Based on this, she builds advice around using routine (to assist procedural memory) and reminder (to provide sensory cues and engender positive feelings). The author asserts that this combination will lead to reward, a ‘win-win’, as both the carer and the person with dementia will feel happy. The book's short chapters each address a different issue in care, such as ‘eating, feeding and nutrition challenges’ and ‘bathing challenges’. Each contains information on the impact of retrogenesis, tips on how to create the best environment and establish a good routine, and worksheets to prompt carers to systematically try different tips to see which helps.

The merits of the book lie in its straightforward expression, positive motivational messages and clear structure, but the advice is somewhat idiosyncratic. References to the evidence base are limited. However, scientifically rigorous studies do not exist to support the practice wisdom of all aspects of dementia care and much of the material that lacks citations would be recognised by experienced dementia care staff as valid. Nevertheless, there are some unusual unfounded assertions, such as ‘Some find purple items more likely to be hoarded, as purple is a sign of royalty’ (p. 118). The emphasis placed on retrogenesis is surprising as this is not widely accepted in dementia care and is somewhat controversial, and there is overemphasis on the use of essential oils and aromatherapy, alongside exaggeration of their effectiveness. ‘Many clinical trials support the effectiveness of essential oils, including lavender and cedar for reducing anxiety and peppermint for mental stimulation’ (p. 35) contrasts starkly with the conclusion of a recent Cochrane review that there is no convincing evidence that aromatherapy or exposure to plant oils is beneficial for people with dementia. In light of this, despite the book's strengths, I would not recommend this as a major source of advice for carers.

